# Uptake of IgG in osteosarcoma correlates inversely with interstitial fluid pressure, but not with interstitial constituents

**DOI:** 10.1054/bjoc.2001.2180

**Published:** 2001-12

**Authors:** C de Lange Davies, B Ø Engesæter, I Haug, I W Ormberg, J Halgunset, C Brekken

**Affiliations:** Department of Physics, Department of Laboratory Medicine, The Norwegian University of Science and Technology, Trondheim, 7491, Norway

**Keywords:** IgG uptake, interstitial fluid pressure, extracellular matrix, vasculature, osteosarcoma

## Abstract

The uptake of therapeutic macromolecules in solid tumours is assumed to be hindered by the heterogeneous vascular network, the high interstitial fluid pressure, and the extracellular matrix. To study the impact of these factors, we measured the uptake of fluorochrome-labelled IgG using confocal laser scanning microscopy, interstitial fluid pressure by the ‘wick-in-needle’ technique, vascular structure by stereological analysis, and the content of the extracellular matrix constituents collagen, sulfated glycosaminoglycans and hyaluronan by colourimetric assays. The impact of the microenvironment on these factors was studied using osteosarcomas implanted either subcutaneously or orthotopically around the femur in athymic mice. The uptake of IgG was found to correlate inversely with the interstitial fluid pressure and the tumour volume in orthotopic, but not subcutaneous tumours. No correlation was found between IgG uptake and the level of any of the extracellular matrix constituents. The content of both collagen and glycosaminoglycans depended on the site of tumour growth. The orthotopic tumours had a higher vascular density than the subcutaneous tumours, as the vascular surface and length were 2–3-fold higher. The data indicate that the interstitial fluid pressure is a dominant factor in controlling the uptake of macromolecules in solid tumours; and the site of tumour growth is important for the uptake of macromolecules in small tumours, extracellular matrix content and vascularization.© 2001 Cancer Research Campaign http://www.bjcancer.com


*British Journal of Cancer* (2001) **85**(12), 1968–1977
© 2001 Cancer Research Campaign
doi: 10.1054/ bjoc.2001.2180, available online at http://www.idealibrary.com on IDEAL

### Uptake of IgG in osteosarcoma correlates inversely with interstitial fluid pressure, but not with interstitial constituents

**C de Lange Davies[1], BØ Engesæter[1], I Haug[1], IW Ormberg[1], J Halgunset[2] and C Brekken[1]**

[1]Department of Physics and [2]Department of Laboratory Medicine, The Norwegian University of Science and Technology, 7491 Trondheim, Norway

**Summary** The uptake of therapeutic macromolecules in solid tumours is assumed to be hindered by the heterogeneous vascular network, the high interstitial fluid pressure, and the extracellular matrix. To study the impact of these factors, we measured the uptake of fluorochrome-labelled IgG using confocal laser scanning microscopy, interstitial fluid pressure by the 'wick-in-needle' technique, vascular structure by stereological analysis, and the content of the extracellular matrix constituents collagen, sulfated glycosaminoglycans and hyaluronan by colourimetric assays. The impact of the microenvironment on these factors was studied using osteosarcomas implanted either subcutaneously or orthotopically around the femur in athymic mice. The uptake of IgG was found to correlate inversely with the interstitial fluid pressure and the tumour volume in orthotopic, but not subcutaneous tumours. No correlation was found between IgG uptake and the level of any of the extracellular matrix constituents. The content of both collagen and glycosaminoglycans depended on the site of tumour growth. The orthotopic tumours had a higher vascular density than the subcutaneous tumours, as the vascular surface and length were 2–3-fold higher. The data indicate that the interstitial fluid pressure is a dominant factor in controlling the uptake of macromolecules in solid tumours; and the site of tumour growth is important for the uptake of macromolecules in small tumours, extracellular matrix content and vascularization. © 2001 Cancer Research Campaign http://www.bjcancer.com

**Keywords**: IgG uptake; interstitial fluid pressure; extracellular matrix; vasculature; osteosarcoma

A major problem using tumour-specific agents such as monoclonal antibodies recognizing tumour-associated antigens and DNA vectors for gene therapy, is the low tumour uptake of the therapeutic agents. The elevated interstitial fluid pressure (IFP) (Jain and Baxter, 1988; Boucher et al, 1990) hinders convection across the capillary wall and through the interstitium. Extravasation of therapeutic agents requires a net transvascular pressure gradient, according to the Starling equation (Kedem and Katchalsky, 1958). However, due to the high permeability of microvessels and lack of functional lymphatics in tumours, the microvascular pressure and the IFP are almost equal (Boucher and Jain, 1992), resulting in a low transvascular pressure gradient.

Macromolecules able to extravasate have to penetrate the interstitium by diffusion and convection (Jain, 2001). The interstitium consists of a protein network embedded in a hydrophilic gel of glycosaminoglycans (GAG) and proteoglycans. It is not clear whether the network of fibrillar collagen or the GAG gel plays the most important role in limiting the interstitial transport of macromolecules. The collagen network which represents the large-scale structure and the structural element of the interstitium, has been assumed to play a minor role for transport, whereas the stabilizing hydrophilic GAG gel has been thought to determine the pore size and thereby regulate the interstitial movement of fluid and macromolecules in tumour tissue (Swabb et al, 1974; Jain, 1987). However, recently the diffusion coefficient and hydraulic conductivity in tumour interstitium were found to correlate with collagen rather than with GAG content (Netti et al, 2000).

The production of extracellular matrix (ECM) macromolecules in the interstitium is a result of an active interaction between the tumour cells and the stromal cells of the host (Gullino and Grantham, 1962; Knudson et al, 1984). The fibroblasts synthesize the ECM constituents in most tumours, whereas the tumour cells regulate the production either directly or indirectly by releasing cytokines that modify the phenotypic expression of the mesenchymal cells of the host (Iozzo, 1985). The composition and structure of ECM as well as parameters such as IFP, vascular structure and vascular permeability are influenced by the microenvironment (Sunderkötter et al, 1994; Fidler, 1995; Fukumura et al, 1998), and thus depending on the site of tumour growth (Fukumura et al, 1997; Hobbs et al, 1998; Brekken et al, 2000a).

Based on all this, we proposed that the tumour uptake of antibodies (IgG) is hindered both by the high IFP and by the ECM composition, and that these parameters are depending on the site of tumour growth. To test this hypothesis, tumour uptake of fluorochrome-labelled IgG was correlated with IFP and the content of ECM constituents in human osteosarcoma xenografts growing either ectopically (subcutaneously) or orthotopically (around femur) in athymic mice. The influence of the microenvironment on the vasculature was assessed by stereological characterization of vascular structure parameters in the ectopic and orthotopic tumour models.

*Received 12 July 2000*
*Revised 3 August 2001*
*Accepted 17 September 2001*

*Correspondence to:* C de L Davies

### MATERIALS AND METHODS

### Tumours

Xenografts were grown in female BALB/c nu/nu mice (Möllergård Breeding Center, Copenhagen, Denmark) in 2 different microenvironments. The orthotopic (o.t.) tumours were initiated by injecting $2 \times 10^6$ human osteosarcoma cells from the cell line OHS (Fodstad et al, 1986) adjacent to the periosteum of both femurs, allowing the tumour to grow around and infiltrate the bones. The subcutaneous (s.c.) tumours were initiated by implanting tumour chunks (~1 $mm^3$) from subcutaneously growing OHS xenografts subcutaneously on both hind legs. The growth curve and histology of the 2 tumour models have been reported previously (Brekken et al, 2000a). Tumour dimensions were measured with a steel calliper. For subcutaneous tumours, the tumour width and length were measured, whereas for orthotopic tumour the 2 widths (width a and width b) of the leg of mouse without tumour and of leg with surrounding tumour were measured. Volumes were estimated using the formula for the volume of a prolate ellipsoid (subcutaneous: $V = \pi/6 \times (\text{width})^2 \times \text{length}$, orthotopic: $V = \pi/6 \times (\text{width a})^2 \times (\text{width b})$). The volume of orthotopic tumours were estimated by subtracting the leg volume of mouse without tumour from the volume of leg with surrounding tumour. The animals were kept under pathogen-free conditions and were allowed food and water ad libitum.

### Experimental procedure

3–5 weeks after tumour implantation, the mice were anasthesized using Hypnorm/Dormicum/sterile water, 1:1:2; 1 ml $kg^{-1}$ body-weight, and the IFP measured using the wick-in-needle method. Fluoresecin-5-isothiocyanate (FITC)-labelled IgG, 20 mg $ml^{-1}$ in 200 μl, was injected in the tail vein immediately after IFP had been measured. The FITC-IgG was allowed to accumulate in the tumour for 24 h. The mice were then sacrificed by cervical dislocation and the tumours cut in 2 parts. Subcutaneous tumours were cut through the centre, and orthotopic tumours along the periosteum. One part was placed in liquid $N_2$ for measurements of IgG uptake. In case of the larger tumours (volume > 400 $mm^3$), the second part was divided between measurements of ECM constituents and estimation of the vasculature, and placed in liquid $N_2$ or fixed in 4% buffered formaldehyde, respectively. In smaller tumours only IFP and ECM constituents were measured.

### Tumour uptake of IgG

The uptake of FITC-labelled IgG was measured using confocal laser scanning microscopy. IgG (rabbit γ-globulin, Sigma, St Louis, MO) was labelled with FITC (Molecular Probes, Eugene, OR) by incubating 2 mg $ml^{-1}$ FITC in DMSO (Sigma, St Louis, MO) per mg IgG for 4 h at room temperature. Bound IgG-FITC was separated from unbound FITC using a PD-10 sepharex G25M column (Amersham Pharmacia Biotech, Buckinghamshire, UK). The ratio between number of FITC molecules per molecule of IgG was approximately 7.

Frozen sections of the tumours, 5 μm thick, were analysed using a 600 MRC (BioRad, Hercules, CA) epi confocal laser scanning microscope (Nikon, Japan). The sections were mounted using Vectashield (Vector Laboratories, Burlingame, CA) to reduced bleaching of the fluorophore. As no other handling of the sections was done, the loss of IgG was assumed to be minimal. The 488 nm argon-laser line was used to excite FITC, and the fluorescence above 520 nm detected. To ensure equally illumination and bleaching of the sections, all sections were only scanned once prior to recording an image, and fluorescent calibration particles (Polysciences, Warrington, PA) were used to control for day-to-day variations in fluorescence intensity.

The fluorescence intensity was quantified by measuring the average pixel intensity (sum of all pixel values per number of pixels) in 4–6 fields across the section, using a 10 × objective. The fluorescence intensity profile from one periphery to the other was thus recorded. Fluorescence intensities of necrotic areas were not measured as IgG often was trapped in such areas. 10 sections approximately 100 μm apart were measured per tumour. IgG uptake was expressed as percentage increase of fluorescence intensity relative to autofluorescence of the tissue.

### Interstitial fluid pressure measurements

IFP was measured using the wick-in-needle technique described in detail elsewhere (Fadnes et al, 1977). Briefly, 4 nylon sutures (6–0 Ethilon; Ethicon Inc, Sommerville, NJ) were placed within a standard 23 gauge hypodermic needle with a side hole 4 mm from the tip. The needle was connected to a pressure transducer (SensoNor, Horten, Norway) via polyethylene tubing (PE-50, Becton-Dickinson, Sparks, MD) filled with sterile heparinized phosphate-buffered saline (PBS) (70 units $ml^{-1}$). Pressures were monitored online using a MacLab analogue to digital data recording system with a sampling rate of 4 Hz (MacLab4/e, ADInstruments, Hastings, UK). Fluid communication between the transducer and the tumour tissue was tested by compression and decompression of the polyethylene tubing, and accepted when the IFP did not differ more than 20% after compression and decompression.

### Disintegration of tissue

The tumour tissue had to be solubilized in order to measure ECM constituents. The excised tumour was cut in pieces and placed in digest buffer (50 mg tissue $ml^{-1}$). The buffer consisted of 125 μg $ml^{-1}$ papain (Sigma, St Louis, MO) in 0.1 M Na-phosphate (Merck, Darmstadt, Germany), 5 mM $Na_2EDTA$ (Merck, Darmstadt, Germany), and 5 mM cysteine-HCl (Sigma, St Louis, MO), pH 6.0. The tumour tissue was finely dispersed with a homogenizer (Polytron; Brinkmann Instrument, Westbury, NY), and incubated in the digest buffer for 18 h at 60˚C.

### GAG content

The total amount of GAG was determined by the reaction of uronic acid with carbazole as described by Bitter and Muir (1962). Briefly, 0.5 ml solubilized tissue diluted (1:3) in water saturated with benzoic acid was carefully layered onto 3 ml of sulfuric acid at 0˚C, shaken, and boiled for 10 min. After cooling to room temperature, 100 μl carbazole (Sigma, St Louis, MO) reagent was added, the samples shaken, boiled for 15 min and cooled to room temperature. The absorbance at 530 nm was measured using a spectrophotometer (model UV-1201; Shimadzu, Kyoto, Japan), and the content of GAG equivalent to uronic acid (UA) was determined from the UA standard curve.

Sulfated GAG (s-GAG) was measured using the Blyscan proteoglycan and s-GAG assay (Biocolor Ltd, Belfast, Ireland),

© 2001 Cancer Research Campaign which is based on the specific binding of the cationic dye 1,9 dimethylmethylene blue (Farndale et al, 1986). Briefly, 100 µl of diluted (1:4) solubilized sample and 1 ml Blyscan dye reagent was mixed for 30 min at room temperature and centrifuged for 10 min. The precipitated polysaccharide-dye complex was dissolved in 1 ml dissociation reagent. The absorbance of bound dye was measured at 656 nm. The s-GAG equivalent to UA was found by measuring the standards of the Blyscan assay with the assay for total GAG.

The content of hyaluronic acid (HA) was estimated as the difference between total GAG and s-GAG, both expressed equivalent to UA.

### Collagen content

The amount of collagen was determined by measuring hydroxyproline according to Woessner (1961). Briefly, 100 µl of solubilized sample was hydrolysed by adding HCl to a final concentration of 6 N. The samples were hydrolysed at 110˚C for 18 h, followed by neutralization using 2.5 N NaOH. Hydroxyproline was oxidated by adding 1 ml chloroamine T (Sigma, St Louis, MO) per 2 ml sample, mixed and left at room temperature for 20 min. Cloroamine T was destroyed by adding 1 ml 3.15 M percloric acid (Merck, Darmstadt, Germany) mixed and left at room temperature for 5 min. Finally, 1 ml p-dimethylaminobenzaldehyde (Sigma, St Louis, MO) was added, mixed and placed at 60˚C for 20 min. The absorbance was measured spectrophotometrically at 557 nm. The concentration of hydroxyproline was determined from the hydroxyproline standard curve, and the content of collagen estimated by assuming 6.94 µg collagen $\mu g^{-1}$ hydroxyproline (Jackson and Cleary, 1976).

### Vascular structure

Formalin-fixed and paraffin-embedded tumours were cut into 5 µm sections. The paraffin was removed by xylene, and the sections rehydrated and trypsin treated (0.1%) (Type II-S, Sigma, St Louis, MO) for 45 min at 37˚C, before blocking against endogenous peroxidase using 3% $H_2O_2$ (Merck, Darmstadt, Germany). The endothelial cells were visualized by staining against CD 31 using the tyramide signal amplification kit (NEN Life Science Products, Boston, MA). Tumour sections were incubated in a humidity chamber overnight at 4˚C with anti-mouse CD31 monoclonal antibody (clone MEC13.3; PharMingingen, San Diego, CA) diluted 1:30, followed by incubation at room temperature for 30 min with biotinylated anti-rat IgG (Vector Lab, Burlinghame, CA) and peroxidase-conjugated streptavidin (NEN Life Science Products, Boston, MA). The amplification procedure included incubation with biotinyl tyramide for 10 min and an additional streptavidin–peroxidase incubation before applying the diaminobenzidine substrate (Sigma, St Louis, MO). The sections were counterstained with haematoxylin (Sigma, St Louis, MO), dehydrated and mounted using Corbit-balsam (Hecht, Germany).

Quantitative information on microvascular structure was obtained using stereological morphometry (Gundersen, 1979; Weibel, 1979). For this purpose, a quadratic counting grid was used. Sections of tumour tissue were examined in a Zeiss light microscope, equipped with a drawing attachment, thus allowing the grid to be visually merged with the image of the tissue. Based on counting performed in the microscope, the microvascular volume, surface and length per unit volume of tumour tissue were determined. 8 sections approximately 100 µm apart, were examined from each tumour, and in each section systematic fields were observed by moving the object stage of the microscope, in 1 mm steps. The stereological parameters were estimated for each tumour by summing the counts over all fields in the section according to the following formulae:

Volume density: $$Vv = \frac{\sum_{s=1}^{n} P_{KS}}{\sum_{s=1}^{n} P_{TS}}$$

Surface density: $$Sv = 2 \times \frac{\sum_{s=1}^{n} I_S}{\sum_{s=1}^{n} L_{TS}}$$

Length density: $$Jv = 2 \times \frac{\sum_{s=1}^{n} N_{KS}}{\sum_{s=1}^{n} A_S}$$

*n* is the number of fields examined in the section. The values for each tumour are the average of 8 sections. $P_{KS}$ is the number of test points hitting the wall or the lumen of a microvessel in any given field, and $P_{TS}$ is the total number of test points hitting any part of the section. $I_S$ is the number of intersections between the test lines and the wall of a microvessel in any given field, and $L_{TS}$ is the total test-line length within the section. $N_{KS}$ is the number of microvessel transections recognized within the grid boundaries in any given field, and $A_S$ is the test area, i.e. the part of the grid area falling within the section. In determining whether or not to count a given vascular profile, the criteria described by Gundersen (1979) were applied.

The stereological formulae are derived based on the assumption that the section thickness is negligible compared with the dimensions of the features of interest, and that the sections constitute a true random sample of the tissue structure. These criteria are considered fulfilled to an acceptable degree as the tissue samples were embedded without any consideration given to their position or orientation in the blocks.

### Statistical analysis

Statistical comparisons of Gaussian data were performed using the one-way analysis of variance, ANOVA. The significance criterion of $P < 0.05$ was used. Correlation analyses were performed by linear regression.

### RESULTS

### Uptake of IgG versus tumour volume and IFP

The uptake of IgG in orthotopic tumours correlated inversely with the tumour volume and with IFP, whereas no such correlation was seen for subcutaneously growing tumours (Figure 1 A, B).

© 2001 Cancer Research Campaign

Correspondingly, the orthotopic tumours showed a positive correlation between tumour volume and IFP. No such correlation was seen for subcutaneously growing tumours (Figure 1C). The correlation between tumour volume and IFP is in accordance with our previous findings (Brekken et al, 2000a).

Comparing the average IgG uptake and IFP for all orthotopic or subcutaneous tumours, the uptake of IgG was found to be independent of the site of tumour growth, whereas orthotopic tumours had about 50% higher IFP than the subcutaneous tumours (Figure 1D). To compare the uptake of IgG between orthotopic and subcutaneous tumours of similar size, the tumours were divided into 3 groups according to their volume. The uptake of IgG was found to be significantly higher in small (< 400 $mm^3$) orthotopic tumours compared with corresponding subcutaneous tumours, whereas no difference was seen for larger tumours (400–1000 $mm^3$ or > 1000 $mm^3$).

### Distribution of IgG in the tissue

The distribution of IgG in the tumours was heterogeneous. Brightest fluorescence intensity was seen around structures resembling blood vessels. A typical example is shown in Figure 2. The bright fluorescent areas stretched approximately 10–70 μm from the vessels, both in orthotopic (panel A, B) and subcutaneous (panel C, D) tumours. In some subcutaneous tumours the fluorescence covered a larger area than seen in orthotopic tumours. The fluorescence intensity was rather weak outside the bright fluorescent area with some scattered fluorescent spots. A higher fluorescence intensity and a higher number of fluorescent areas were seen in the periphery (panel A and C) compared to the central part (panel B and D) of some of the tumours (Figure 2). The majority of the tumours, (70% of the orthotopic and 85% of the subcutaneous tumours), however, had weak fluorescence intensity throughout the sections and no differences in fluorescence intensity between the periphery and central parts of the tumours could be detected.

### Content of ECM versus IgG uptake, tumour volume and IFP

To study the impact of the ECM composition on the uptake of macromolecules, the content of collagen, total GAG, s-GAG, and HA were measured in each tumour. No correlation between IgG uptake and any of the ECM constituents was seen for either of the 2 tumour models (Figure 3).

Regression analysis of the content of the ECM constituents versus tumour volume (Figure 4) or IFP (data not shown) were done. Collagen content decreased with increasing tumour volume for orthotopic tumours, whereas a positive correlation was seen for subcutaneous tumours. No correlation between total GAG content and the tumour volume was found. However, s-GAG increased with increasing volume of orthotopic tumours, and correspondingly HA decreased. No correlation between IFP and any of the ECM constituents was seen.

### Content of ECM and the site of tumour growth

Comparing the average content of ECM constituents in orthotopically and subcutaneously growing tumours, significant differences were found (Figure 5). Orthotopic tumours had a significantly lower level of total GAG, a higher level of s-GAG and a correspondingly lower level of HA compared with subcutaneous

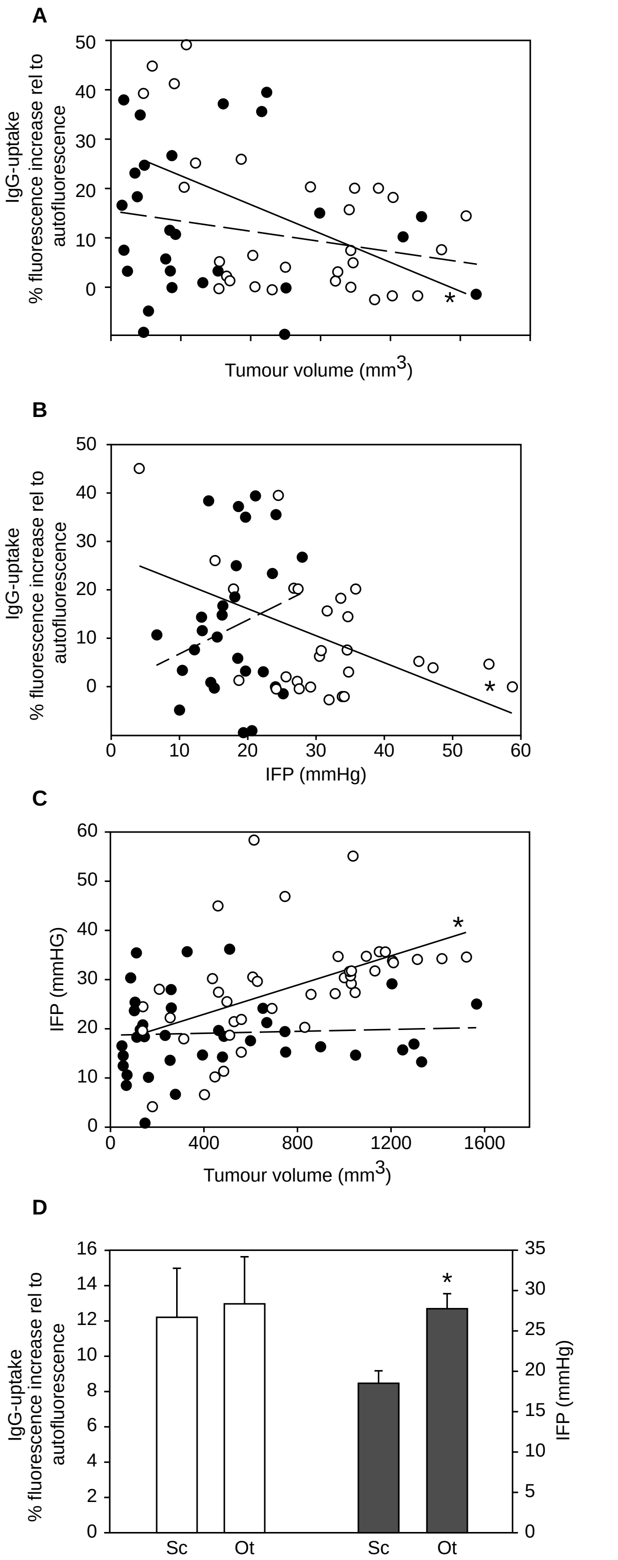

**Figure 1** Uptake of IgG in orthotopic (○) (solid line) and subcutaneous (●) (dashed line) xenografts as a function of tumour volume (**A**) and IFP (**B**). IFP in orthotopic (○) (solid line) and subcutaneous (●) (dashed line) xenografts as a function of tumour volume (**C**). The curves were fitted to the data by linear regression. The average uptake of IgG (open bars) (*n* = 30–39 tumours) and average IFP (filled bars) (*n* = 39–42 tumours) in all orthotopic and subcutaneous xenografts. Standard errors are indicated. *Indicates significant correlation or difference

© 2001 Cancer Research Campaign

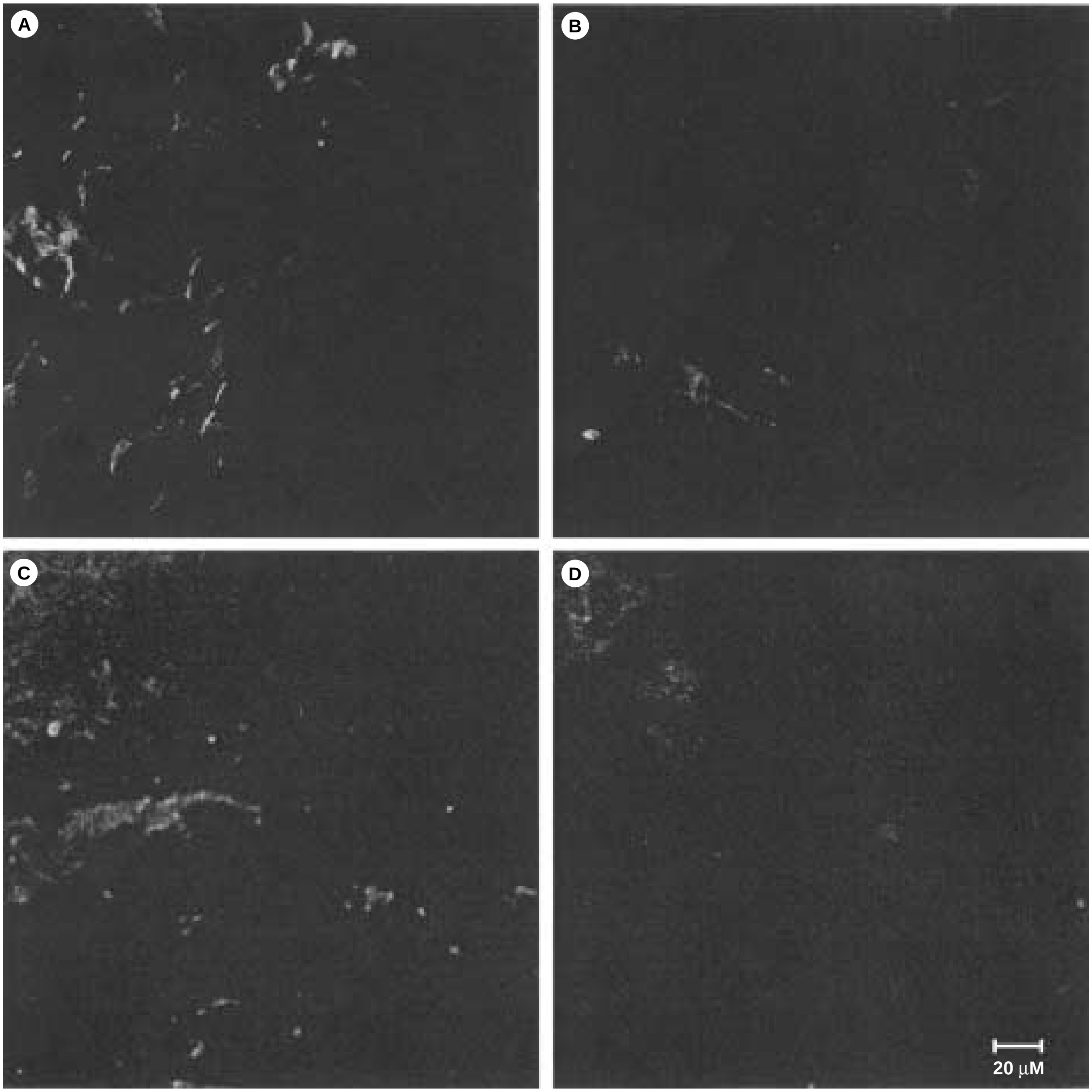

**Figure 2** Distribution of IgG-FITC in the periphery (**A**,**C**) and central part (**B**,**D**) of orthotopic (**A**,**B**) and subcutaneous (**C**,**D**) xenografts. Bar = 20 µm

**Table 1** Content of collagen, total GAG, s-GAG and HA in normal and tumour tisssue[1]

| Tissue | Collagen (µg/mg) | Tot GAG (µg UA eqv/mg) | s-GAG (µg UA eqv/mg) | HA (µg UA eqv/mg) |
|---|---|---|---|---|
| Skin | 27.33 ± 8.06 | 6.90 ± 0.36 | 1.09 ± 0.09 | 5.82 ± 0.39 |
| Muscle | 2.84 ± 0.17 | 2.33 ± 0.16 | 0.38 ± 0.02 | 1.95 ± 0.16 |
| Sc ostosarcoma | 1.57 ± 0.11 | 1.19 ± 0.03 | 0.44 ± 0.02 | 0.71 ± 0.04 |
| Ot osteosarcom | 2.04 ± 0.18 | 1.04 ± 0.03 | 0.71 ± 0.02 | 0.34 ± 0.04 |

[1]GAG, s-GAG and HA was measured equivalent to UA per mg wet tissue tumours (Figure 5A). The collagen content was significantly higher in orthotopic tumours (Figure 5B). The ECM constituents in the periphery and central parts of the tumour were compared. Only s-GAG showed a significantly higher level in central parts of orthotopically growing tumours. Collagen, total GAG or HA showed no such variations (data not shown).

The content of the ECM constituents was found to be lower in tumour tissue compared with normal tissue (Table 1). Collagen and

 *© 2001 Cancer Research Campaign*

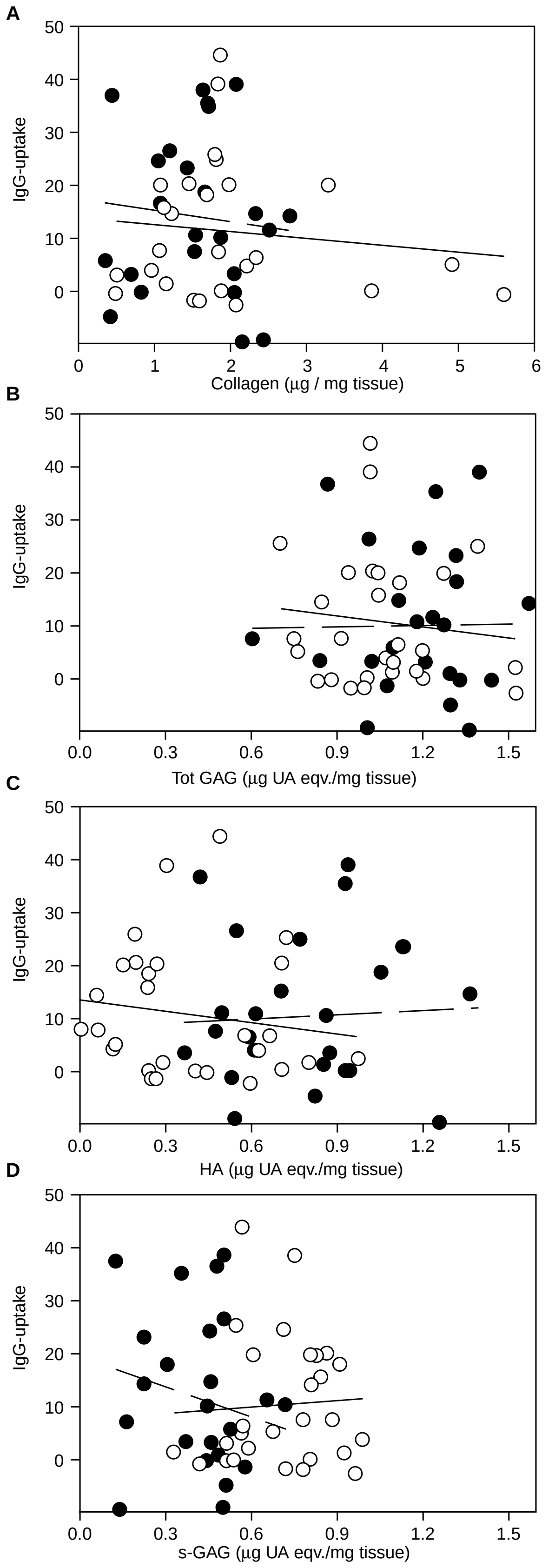

**Figure 3** Uptake of IgG in orthotopic (○) (solid line) and subcutaneous (●) (dashed line) xenografts as a function of content of collagen (**A**), total GAG (**B**), HA (**C**), and s-GAG (**D**)

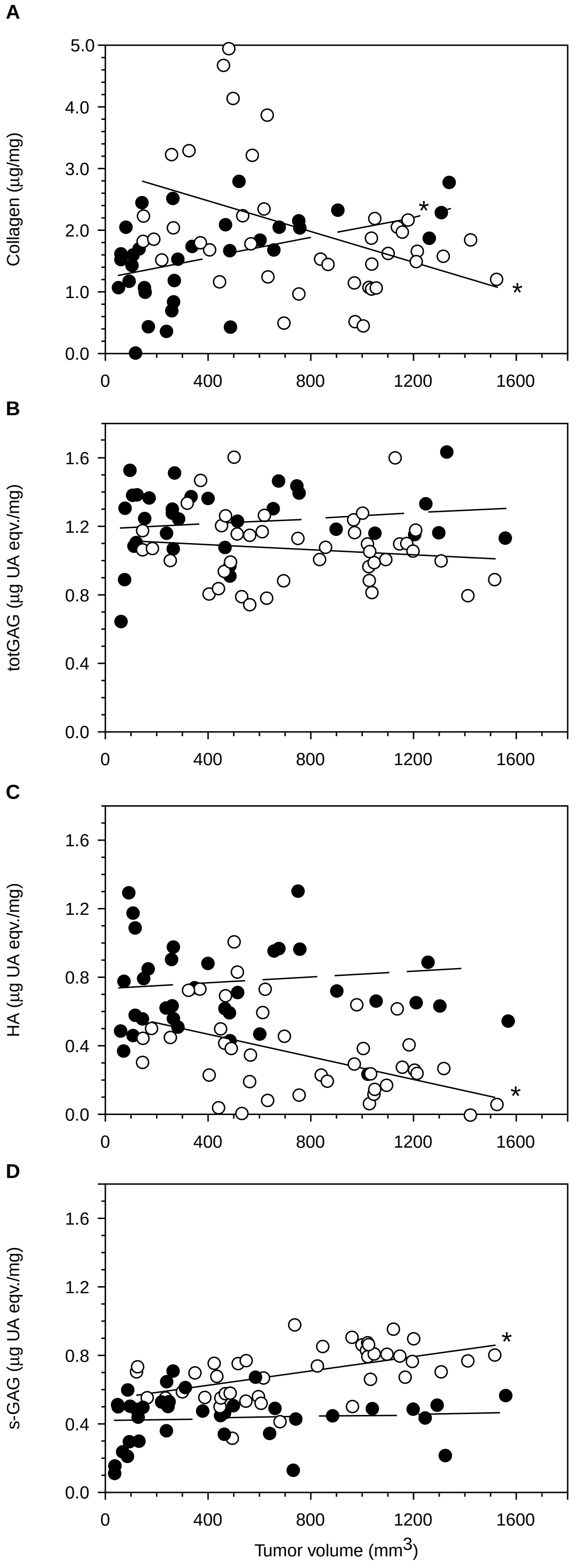

**Figure 4** Content of collagen (**A**), total GAG (**B**), HA (**C**), and s-GAG (**D**) in orthotopic (○) (solid line) and subcutaneous (●) (dashed line) xenografts as a function of tumour volume. *Indicates significant correlation

*© 2001 Cancer Research Campaign*

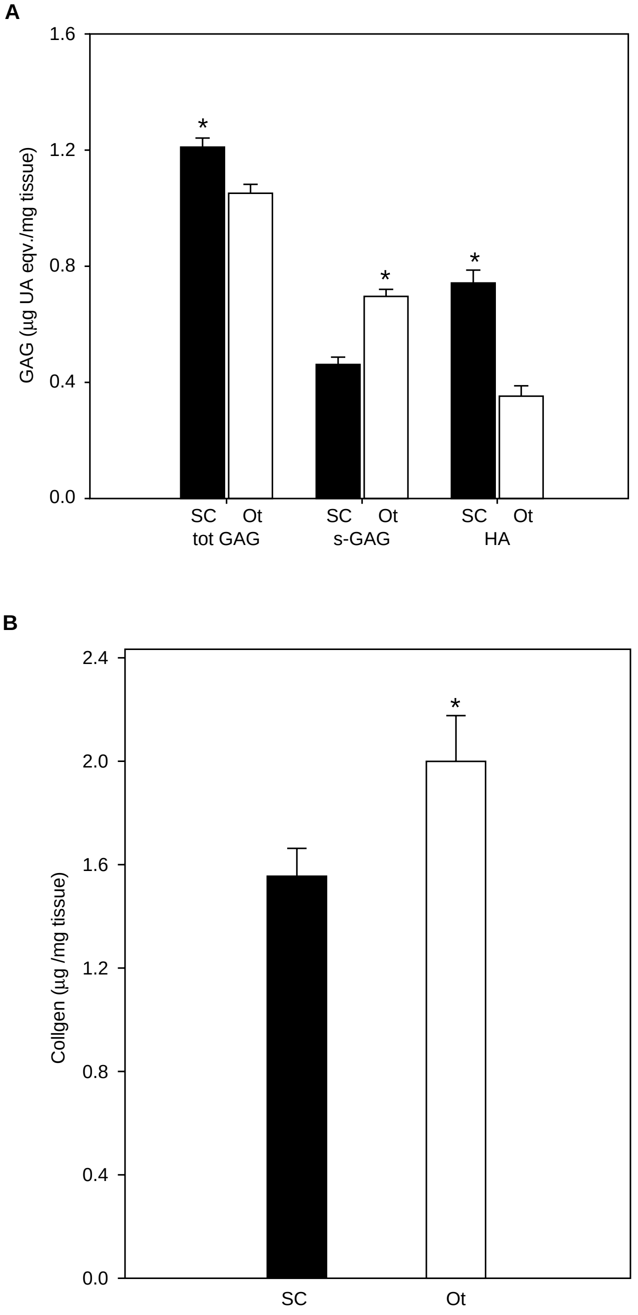

**Figure 5** Average content of GAG both total GAG, s-GAG and HA (**A**), and collagen (**B**) in subcutaneous (filled bars) and orthotopic (open bars) xenografts. Each value is the mean of 36–42 tumours. Standard errors are indicated. *Indicates significant difference between orthotopic and subcutaneous xenografts total GAG were approximately 15- and 6-fold lower in tumours than in skin. The difference between muscle and tumours was smaller.

### Uptake of IgG and vascularization

To study the influence of the microenvironment on the vascular structure, vascular volume, surface and length were estimated by stereological analysis. Subcutaneous tumours were less vascularized than orthotopic tumours, demonstrated by approximately 2- and 3-fold lower vascular surface and vascular length densities

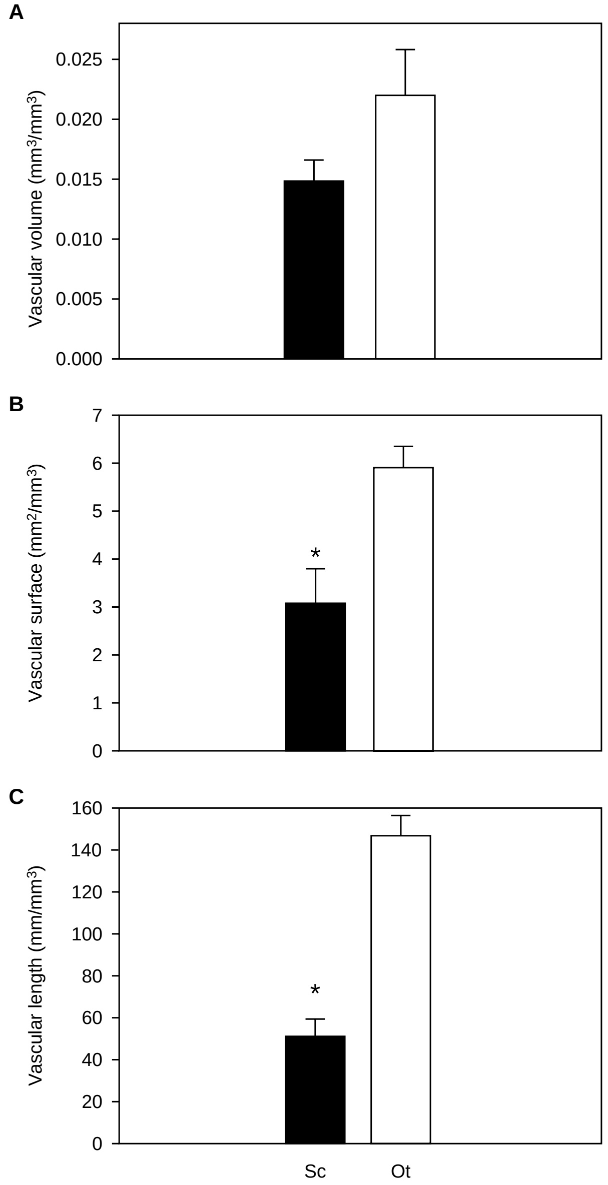

**Figure 6** Vascular structure estimated by stereology. Vascular volume (**A**), vascular surface (**B**) and vascular length (**C**) in orthotopic (open bars) and subcutaneous (filled bars) xenografts. Each value is the mean of 6–8 tumours. Standard errors are indicated. *Indicates significant difference between orthotopic and subcutaneous xenografts

(Figure 6). The vascular volume seemed to be smaller in subcutaneous tumours than orthotopic, but the difference was not significant. Regression analysis of IgG uptake and the 3 vascular parameters did not show any correlation (data not shown).

### DISCUSSION

### Correlation between uptake of IgG and IFP depends on the site of tumour growth

The high IFP seems to be the most dominant factor in limiting the uptake of macromolecules in orthotopically growing osteosarcoma xenografts. Tumour uptake of IgG correlated inversely with IFP, and with the volume of orthotopic tumours, but not with the ECM constituents. The increased IgG uptake in

© 2001 Cancer Research Campaign small orthotopic tumour is probably caused by an increased transvascular flux, thus the low IFP in small orthotopic tumours indicates increased transvascular flux. The enhanced IFP is driven by the microvascular pressure, and the hydrostatic pressure gradient across the vascular wall in tumours is normally very low (Boucher and Jain, 1992). However, in the periphery of the tumour, IFP decreases and the transvascular flux is higher than in the central parts of the tumour (Jain and Baxter, 1988). Therefore, the increased uptake of IgG might take place in the periphery of the tumour. This is consistent with the images of the distribution of IgG in small tumours (Figure 2) which showed a higher uptake of IgG in the periphery than in the central parts of the tumour. Fluorescent IgG was observed around structures resembling vessels indicating that IgG was able to penetrate approximately 10–70 μm into the interstitium. The high IFP might impede further penetration. Enhanced macromolecule uptake in tumour tissue has been demonstrated by periodic cycling the IFP using hyaluronidase (Brekken et al, 2000b) or angiotensin II (Netti et al, 1999), thereby increasing the transvascular flux and uptake of antibody. Also, lowering the IFP using prostaglandin $E_1$ has been shown to increase the transvascular transport and tumour uptake of low-molecular-weight molecules (Rubin et al, 2000).

The correlation between IgG uptake and IFP might reflect changes in vessel permeability, as the high interstitial fluid pressure is caused by the hyperpermeability of tumour vessels (Boucher and Jain, 1992; Yuan, 1998). However, there is no clear relationship between vascular permeability and tumour volume. Reducing the tumour volume by anti-VEGF antibody reduces the vascular permeability, as well as IFP (Lee et al, 2000). Thus, a lower vascular permeability (but sufficient for IgG) associated with a smaller tumour volume might induce a lower IFP and thereby a higher uptake of macromolecules, as seen in the present work. On the other hand, the volume of human melanomas are reported to either show no correlation with vascular permeability, or an inverse correlation (Bjørknæs and Rofstad, 2001). This is consistent with the lack of correlation between IgG uptake, IFP and volume of subcutaneous tumours.

The correlation between macromolecule uptake and IFP depended on the site of tumour growth. This is probably due to differences in the microenvironment influencing parameters important for macromolecule uptake. The vascular permeability is reported to depend on the microenvironment (Fukumura et al, 1997). It is assumed that the balance of interactions between cytokines released by host stromal cells and tumour cells determines the vascular permeability (Yuan, 1998). The growth curves of the 2 tumour models were almost the same (Brekken et al, 2000a), indicating that the site-dependent correlation between IgG uptake, IFP and tumour volume, was not due to differences in growth rate.

### No correlation between uptake of IgG and content of ECM

Penetration of molecules through the interstitium is characterized by the hydraulic conductivity and the diffusion coefficient. The interstitial hydraulic conductivity is reported to correlate inversely with GAG (Swabb et al, 1974), and in a mathematical model found to affect the steepness of the IFP profile in the tumour periphery (Jain and Baxter, 1988). However, within the range of GAG measured in the OHS tumours (0.7–1.6 μg UA equivalents per mg tissue) no significant correlation between GAG (total GAG, s-GAG, HA) and IFP or GAG and IgG uptake was seen. A possible increase in hydraulic conductivity with decreasing GAG might thus not be sufficient to increase the interstitial filtration of fluid and extravasation of IgG.

The high IFP might render convection impossible, thus diffusion becomes the only transport mechanism. The diffusion coefficient is found to correlate inversely with the amount of collagen in tumour tissue (Netti et al, 2000). The collagen content in the OHS tumours varied a factor 10 (0.5–5 μg $mg^{-1}$ tissue), but even if the diffusion coefficient increased with decreasing collagen content, it did not have any major impact on the IgG uptake, probably due to the low diffusion coefficient of such a large molecule.

Diffusion and convection are probably not only depending on the content of the ECM constituents, as the assembly and structure of the ECM also play an important role (Pluen et al, 2001). Tumours with a tight collagen network are more resistant to macromolecular penetration than tumours with a loose collagen network. Comparing the diffusion coefficient in tumours growing either in dorsal chambers or in cranial windows, Pluen et al (2001) found a lower diffusion coefficient in tumours in dorsal windows associated with a higher level of collagen organized into fibrils. The collagen network stabilizes the GAG gel, and such stabilizing is assumed to increase the hindrance of macromolecules through the ECM (Netti et al, 2000). In accordance with this suggestion, collagen gels in vitro are found to exhibit resistance to convection which is significantly less than in tissue having the same content of collagen (Jackson et al, 1991; Saltzman et al, 1994). Also gels consisting of a combination of collagen and HA have a higher resistance to macromolecular diffusion than gels of either collagen or HA alone (Shenoy and Rosenblatt, 1995). Degradation of collagen or the GAG gel will induce a structural change and remodelling of the ECM, affecting physiological properties of the tissue. Collagenase is reported to increase the diffusion coefficient of IgG in tumour tissue (Netti et al, 2000), whereas hyaluronidase reduces the diffusion of albumin in lung interstitium (Qiu et al, 1999).

### ECM content depends on the site of tumour growth

The content of collagen depended on the tumour volume, and the correlation between collagen and tumour volume as well as the average content of collagen depended on the site of tumour growth. Collagen decreased or increased with the volume of orthotopically or subcutaneously growing tumours, respectively. This difference might be due to organ-specific fibroblasts influencing the production of collagen and collagenase as the tumours are growing. Co-cultures of colon fibroblasts and colon carcinoma cells are shown to produce collagenase IV, whereas skin fibroblasts and colon carcinoma cells did not (Fabra et al, 1992). A higher density of host stromal cells has been found in tumours with a high level of collagen organized into fibrils growing subcutaneously in dorsal chambers compared with the same tumours grown in cranial windows where the collagen was less organized (Pluen et al, 2001).

The amount of total GAG was independent of the tumour volume whereas HA and s-GAG depended on the volume of orthotopically growing tumours. The amount of total GAG, HA and s-GAG depended on the site of tumour growth. This might indicate that either the fibroblasts or the interaction between the osteosarcoma cells and the fibroblasts increased the synthesis of GAG and HA in subcutaneously growing tumours. In accordance with this, co-cultures of skin fibroblasts and lung carcinoma cells

© 2001 Cancer Research Campaign 

have been shown to increase the synthesis of HA compared with the 2 cell types alone, and contact between tumours cells and fibroblasts appears to be required (Knudson et al, 1984).

The amount of the ECM constituents was in the same range as found in other human xenografts and murine tumours (Netti et al, 2000). Tumour tissue had lower level of collagen and GAG than found in normal tissue, probably due to higher activity of degradation enzymes (Jain, 1987).

### Vascularization depends on the site of tumour growth

The orthotopic tumours had a higher vascular density than the subcutaneous tumours, demonstrated by a 2–3-fold increase in the vascular surface and vascular length per tumour volume. The difference probably reflects a lower number of vessels and an increase in vascular diameter in subcutaneously growing tumours compared with orthotopic tumours, and is consistent with the more pronounced necrosis observed in the subcutaneous OHS tumours compared to the orthotopic tumours (Brekken et al, 2000a). The vascular volume, surface and length were in the same range as found for human melanomas (Solesvik et al, 1982) or mammary adenocarcinoma (Hilmas and Gillette, 1974). No correlation between the vascular parameters and IgG uptake was seen. This might be because the smallest tumours ($< 400\ mm^3$) were not included in the analysis of the vascular structure.

Tumour angiogenesis is induced by the interaction between tumour cells and stromal cells of the host, thereby modulating the production of various cytokines such as the angiogenic factor VEGF (Fukumura et al, 1997) or the anti-angiogenic factor TGF-β1 (Gohongi et al, 1999). The vascularization might thus depend on the microenvironment, and whether implantation at an orthotopic or ectopic growth site induces angiogenesis, is tumour-dependent. Both orthotopic (Fidler, 1995) and subcutaneous (Fukumura et al, 1997; Bernsen et al, 1999) tumours are reported to be highly vascularized. The higher vascular density of orthotopic OHS xenograft should imply a higher uptake of IgG in orthotopic than in subcutaneous tumours. However, even though IgG was well distributed throughout the vascular network, transvascular transport and penetration of IgG through the interstitium might be limited by the high IFP. This might explain why no difference was seen in IgG uptake between orthotopic and subcutaneous tumours larger than $400\ mm^3$. Another explanation might be that the enhanced IFP and higher vascular density of orthotopic tumours compared with subcutaneous tumours, will respectively limit and favour IgG uptake, resulting in no difference in IgG uptake in the 2 tumour models.

### Clinical implications

The present data indicate that lowering the IFP and thereby inducing a transvascular pressure gradient, is an efficient transport parameters to modulate in order to increase the uptake of therapeutic macromolecules. Lowering the IFP might have a more profound clinical impact than modulating the content of the ECM constituents. However, the present results can not exclude the significance of the ECM structure. The assembly and structure of the ECM might be more important in determining the interstitial transport than the level of the constituents themselves.

### ACKNOWLEDGEMENTS

The authors gratefully acknowledge the technical assistance of Inger Beate Følstad. We are indebted to Kristin Bringedal, Dept of Pathology, University Hospital of Trondheim, for preparing paraffin- and frozen-tissue sections.

This work was supported by the Norwegian Cancer Society.

### REFERENCES

Bernsen HJJA, Rijken PFJW, Hagenmeier NEM and van der Kogel AJ (1999) A quantitative analysis of vascularization and perfusion of human glioma xenografts at different implantation sites. *Microvasc Res* **57**: 244–257

Bitter T and Muir HM (1962) A modified uronic acid carbazole reaction. *Anal Biochem* **4**: 330–334

Bjørnæs I and Rofstad EK (2001) Microvascular permeability to macromolecules in human melanoma xenografts assessed by contrast-enhanced MRI – intertumor and intratumor heterogeneity. *Magn Reson Imaging* (in press)

Boucher Y and Jain RK (1992) Microvascular pressure is the principal driving force for interstitial hypertension in solid tumors: Implications for vascular collapse. *Cancer Res* **52**: 5110–5114

Boucher Y, Baxter LT and Jain RK (1990) Interstitial pressure gradients in tissue-isolated and subcutaneous tumors: Implications for therapy. *Cancer Res* **50**: 4478–4484

Brekken C, Bruland ØS and Davies CdeL (2000a) Interstitial fluid pressure in human osteosarcoma xenografts: Significance of implantation site and the response to intratumoral injection of hyaluronidase. *Anticancer Res* **20**: 3503–3512

Brekken C, Hjelstuen MH, Bruland ØS and Davies CdeL (2000b) Hyaluronidase-induced periodic modulation of the interstitial fluid pressure increases selective antibody uptake in human osteosarcoma xenografts. *Anticancer Res* **20**: 3513–3520

Fabra A, Nakajima M, Bucana CD and Fidler IJ (1992) Modulation of the invasive phenotype of human colon carcinoma cells by organ specific fibroblasts of nude mice. *Differentiation* **52**: 101–110

Fadnes HO, Reed RK and Aukland K (1977) Interstitial fluid pressure in rats measured with a modified wick technique. *Microvasc Res* **14**: 27–36

Farndale RW, Buttle DJ and Barrett AJ (1986) Improved quantitation and discrimination of sulphated glycosaminoglycans by use of dimethylmethylene blue. *Biochim Biophys Acta* **883**: 173–177

Fidler IJ (1995) Modulation of the organ microenvironment for treatment of cancer metastasis. *J Natl Cancer Inst* **87**: 1588–1592

Fodstad Ø, Brøgger A, Bruland Ø, Solheim ØP, Nesland JM and Pihl A (1986) Characteristics of a cell line established from a patient with multiple osteosarcoma, appearing 13 years after treatment for bilateral retinoblastoma. *Int J Cancer* **38**: 33–40

Fukumura D, Yuan F, Monsky WL, Chen Y and Jain RK (1997) Effect of host microenvironment on the microcirculation of human colon adenocarcinoma. *Am J Pathol* **151**: 679–688

Fukumura D, Xavier R, Sugiura T, Chen Y, Park E-C, Lu N, Selig M, Nielsen G, Taksir T, Jain RK and Seed B (1998) Tumor induction of VEGF promotor activity in stromal cells. *Cell* **94**: 715–725

Gohongi T, Fukumura D, Boucher Y, Yun C-O, Soff GA, Compton C, Todoroki T and Jain RK (1999) Tumor-host interactions in the gallbladder suppress distal angiogenesis and tumor growth: Involvement of transforming growth factor β1. *Nat Med* **5**: 1203–1208

Gullino PM and Grantham FH (1962) The influence of the host and the neoplastic cell population on the collagen content of a tumor mass. *J Natl Cancer Inst* **27**: 648–653

Gundersen HJ (1979) Estimations of tubuli or cylinder Lv, Sv, and Vv on thick sections. *J Microsc* **117**: 333–345

Hilmas DE and Gillette EL (1974) Morphometric analysis of the microvasculature of tumors during growth and after irradiation. *Cancer* **33**: 103–110

Hobbs SK, Monsky WL, Yuan F, Roberts WG, Griffith L, Torchilin VP and Jain RK (1998) Regulation of transport pathways in tumor vessels: Role of tumor type and microenvironment. *Proc Natl Acad Sci USA* **95**: 4607–4612

Iozzo R (1985) Biology of disease. Proteoglycans: Structure, function, and role in neoplasia. *Lab Invest* **53**: 373–396

*© 2001 Cancer Research Campaign*

Jackson DS and Cleary EG (1976) The determination of collagen and elastin. In: Glick D (ed.) Methods of biochemical analysis. Interscience publ., New York, pp 25–76

Jackson RL, Bush SJ and Cardin AD (1991) Glycosaminoglycans: molecular properties, protein interactions, and role in physiological processes. *Physiol Rev* **71**: 481–539

Jain RK (1987) Transport of molecules in the tumor interstitium: A review. *Cancer Res* **47**: 3039–3051

Jain RK (2001) Delivery of molecular and cellular medicine to solid tumors. *Advanced Drug Delivery Reviews* **46**: 149–168

Jain RK and Baxter LT (1988) Mechanisms of heterogeneous distribution of monoclonal antibodies and other macromolecules in tumors: Significance of elevated interstitial pressure. *Cancer Res* **48**: 7022–7032

Kedem O and Katchalsky A (1958) Thermodynamic analysis of the permeability of biological membranes to non-electrolytes. *Biochim Biophys Acta* **27**: 229–245

Knudson W, Biswas C and Toole BP (1984) Interactions between human tumor cells and fibroblasts stimulate hyaluronate synthesis. *Proc Natl Acad Sci USA* **81**: 6767–6771

Lee C-G, Heijn M, di Tomaso E, Griffon-Etienne G, Ancukiewicz M, Koike C, Park KR, Ferrara N, Jain RK, Suit HD and Boucher Y (2000) Anti-vascular endothelial growth factor treatment augments tumor radiation response under normoxic or hypoxic conditions. *Cancer Res* **60**: 5565–5570

Netti PA, Hamberg LM, Babich JW, Kierstead D, Graham W, Hunter GJ, Wolf GL, Fischman A, Boucher Y and Jain RK (1999) Enhancement of fluid filtration across tumor vessels: Implication for delivery of macromolecules. *Proc Natl Acad Sci USA* **96**: 3137–3142

Netti PA, Berk DA, Swartz MA, Grodzinsky AJ and Jain RK (2000) Role of extracellular matrix assembly in interstitial transport in solid tumors. *Cancer Res* **60**: 2497–2503

Pluen A, Boucher Y, Ramanujan S, McKee TD, Gohongi T, di Tomaso E, Brown EB, Izumi Y, Campbell RB, Berk DA and Jain RK (2001) Role of tumor-host interactions in interstitial diffusion of macromolecules: Cranial vs. subcutaneous tumors. *Proc Natl Acad Sci USA* **98**: 4628–4633

Qiu XL, Brown LV, Parameswaran S, Marek VW, Ibbott GS and Lai-Fook SJ (1999) Effect of hyaluronidase on albumin diffusion in lung interstitium. *Lung* **177**: 273–288

Rubin K, Sjöquist M, Gustafsson AM, Isaksson B, Salvessen G and Reed RK (2000) Lowering of tumoral interstitial fluid pressure by prostaglandin $E_1$ is paralleled by an increased uptake of $^{51}$Cr-EDTA. *Int J Cancer* **86**: 636–643

Saltzman WM, Radomsky ML, Whaley KJ and Cone RA (1994) Antibody diffusion in human cervical mucus. *Biophys J* **66**: 508–515

Shenoy V and Rosenblatt J (1995) Diffusion of macromolecules in collagen and hyaluronic acid, rigid-rod – flexible polymer, composite matrices. *Macromolecules* **28**: 8751–8758

Solesvik OV, Rofstad EK and Brustad T (1982) Vascular structure of five human malignant melanomas grown in athymic nude mice. *Br J Cancer* **46**: 557–567

Sunderkötter C, Steinbrink K, Goebeler M, Bhardwaj R and Sorg C (1994) Machrophages and angiogenesis. *J Leukoc Biol* **55**: 410–422

Swabb EA, Wei J and Gullino PM (1974) Diffusion and convection in normal and neoplastic tissue. *Cancer Res* **34**: 2814–2822

Weibel ER (1979) Stereological Methods. Vol I. Practical Methods. Academic Press, New York

Woessner JF (1961) The determination of hydroxyproline in tissue and protein samples containing small proportions of this amino acid. *Arch Biochem Biophys* **93**: 440–447

Yuan F (1998) Transvascular drug delivery in solid tumors. *Sem Radiat Oncol* **8**: 164–175

© 2001 Cancer Research Campaign
